# ToF-SIMS and AFM Characterization of Brown Cosmetic Contact Lenses: From Structural Analysis to the Identification of Pigments

**DOI:** 10.1155/2020/6134627

**Published:** 2020-01-22

**Authors:** Seon Hee Kim, Jihye Lee, Yun Jung Jang, Kang-Bong Lee, Yeonhee Lee

**Affiliations:** ^1^Advanced Analysis Center, Korea Institute of Science & Technology, Seoul 02792, Republic of Korea; ^2^Department of Materials Science and Engineering, Korea University, Seoul 02841, Republic of Korea; ^3^National Agenda Research Division, Korea Institute of Science & Technology, Seoul 02792, Republic of Korea

## Abstract

Over the years, soft contact lenses for vision correction and cosmetic and therapeutic purposes have been greatly improved. For cosmetic contact lenses, the pigments need to be nontoxic, and the position of the pigment layer is particularly important because of the risks posed by pigment elution and the roughness of the lens surface. In this paper, we characterized the properties of brown cosmetic contact lenses made by three different manufacturers using surface analytical techniques. The surface topographies of the noncolored and colored parts were obtained by atomic force microscopy (AFM), and the position and composition of the pigment layer were determined by analyzing the cross section of the contact lenses using scanning electron microscopy with energy-dispersive X-ray spectroscopy (SEM-EDX). The influence of pigment location on surface roughness was also examined. In addition, to find the method of the evaluation for the risk of surface elution of the pigments in the colored parts, the mass spectra and ion images of the surfaces were obtained by time-of-flight secondary ion mass spectrometry (ToF-SIMS) with a new sample preparation. From the ToF-SIMS spectra, we observed specific fragment ions of the poly(hydroxyethyl methacrylate) (PHEMA) polymer and found differences in the composition of the pigment layer depending on the manufacturers. The cross-sectioned image and 3D chemical characterizations of metallic and specific ions in the brown cosmetic contact lenses clearly indicated the spatial distribution and location of the pigment layer that can be used for the evaluation of pigment elution.

## 1. Introduction

Daily disposable soft contact lenses based on hydroxyethyl methacrylate (HEMA) have become increasingly popular. These lens materials are composed of copolymers of HEMA and other hydrophilic or lipophilic monomers such as *N*-vinyl pyrrolidone (NVP) and methacrylates, the diversity of which contributes to the wide range of water contents of both ionic and nonionic lens materials [1, 2]. Among these contact lenses, cosmetic-type soft lenses containing colored patterns are rapidly gaining market share over the conventional clear-type soft lenses among young people, especially in Asian markets [3, 4]. Cosmetic contact lenses are used to change or enhance the iris color to give a softer look through complex print patterns or a pigmented ring pattern that overlap with the limbal ring of the iris [5]. However, the presence of pigments may alter the surface properties and affect the wearing comfort of these pigmented lenses compared with clear contact lenses. Many manufacturers have showcased their own printing technologies to produce smooth pigmented lenses. The pigment layer is located on the front or on the back surface of the lens but can also be contained within the lens matrix depending on the manufacturing method [6–9]. If any pigments are released on the lens surface, they can contact with the cornea or with the ocular surface and could potentially compromise the ocular health. Recent studies demonstrated that lenses with pigments on the surface resulted in greater increase in ocular inflammation [5]. Thus, recent studies have shown that leakage of pigments from the lens can cause eye irritation, and the surface roughness caused by the pigment layer increases the adhesion rate of bacteria on the contact lens surface, potentially leading to eye infection [10–18].

The advancements in technology have enabled the information concerning the surface properties of contact lenses to be obtained at a more microscopic level. Several techniques have been reported in the literature to characterize lens materials and monitor the stability of the pigment layers, such as optical microscopy, optical coherence tomography, scanning electron microscopy (SEM), SEM with energy-dispersive X-ray spectroscopy (SEM-EDX), atomic force microscopy (AFM), and focused ion beam (FIB), [19–25]. Some researchers showed the surface changes by AFM after daily wear to investigate the wearing effect of the lens [26, 27]. Surface techniques such as X-ray photoelectron spectroscopy (XPS) and time-of-flight secondary ion mass spectrometry (ToF-SIMS) were also used to analyze the pigment distribution as well as lens materials [28–35]. Among the surface analytical techniques, AFM is a well-established technique for flatness analysis and imaging of biomaterial surfaces so that it has been often used to analyze the roughness of various soft contact lenses [21–24]. The surface roughness of a cosmetic contact lens has the potential to impact ocular physiology and product performance. Lau et al. measured the coefficient of friction (CoF) by using a microtribometer and compared the surface roughness of five cosmetic contact lenses using AFM to investigate the potential impact on comfort since CoF has been associated with end-of-day comfort performance [22].

Above all, ToF-SIMS has been used to obtain the surface chemical information of contact lenses [30–35]. It has previously been used to investigate soft contact lens materials, providing chemical information on the surface. It is also able to provide ion images of the elemental and molecular composition of contact lens surfaces. However, previous studies have not shown the 3D chemical imaging for pigment distribution of the cosmetic contact lens that will be useful to determine the diffusion of the pigment layer. And there is a limitation to have ion images of the cross-sectioned lens using ToF-SIMS because of its tricky sample preparation method.

In this study, HEMA-based brown cosmetic contact lenses made by three different manufacturers were analyzed using surface analysis techniques such as AFM, SEM-EDX, and ToF-SIMS. AFM analysis was performed to investigate the effect of the pigment layer on the roughness of the front surface or the back surface. The locations of the pigment layer in the cross-sectioned lenses were identified by SEM-EDX and ToF-SIMS with a new sample preparation method. The 3D chemical mapping of the cosmetic lenses was newly achieved using ToF-SIMS to determine the position and spatial distribution of the pigment layer in the colored part.

## 2. Experimental Section

### 2.1. Materials and Sample Preparation

Three commercially available brown cosmetic contact lenses made of poly(2-hydroxyethyl methacrylate) (PHEMA), representing the three most popular brands in Korea, were used in this study: Acuvue (Johnson & Johnson Vision Care, Ireland, Lens CCL-A), Bausch + Lomb (Bescon Co., Ltd., Korea, Lens CCL-B), and Clalen (Interojo, Inc., Korea, Lens CCL-C). To reduce the number of variables, we chose only brown among a wide range of lens colors. The properties of each cosmetic contact lens are summarized in Table 1.

The contact lens samples were prepared according to the requirements of each analytical instrument. However, lenses made of soft polymers are rich in moisture, and if dried, the original characteristics of the contact lens, such as thickness and hardness, may change. In particular, when analysis is performed under ultrahigh vacuum equipment, which is vulnerable to moisture, it is necessary to cut the sample into the smallest pieces possible for analysis and then make the measurements rapidly while maintaining the lens shape as much as possible. All of the samples were measured within 2 hours after sample preparation to preserve their original properties.

**Figure 1:**
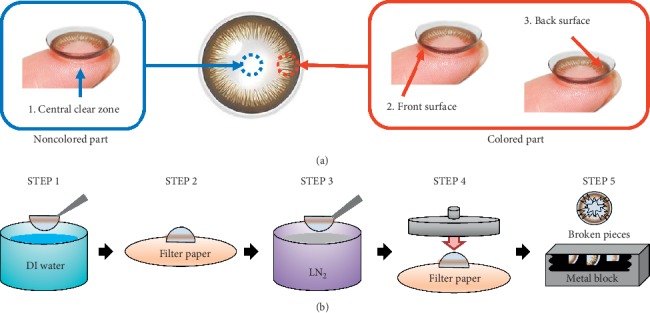
Schematic drawing of (a) analysis area of a cosmetic lens and (b) preparation procedure of a cross-sectioned sample.

To minimize the effect of the preservative solution on the surface analysis, contact lens samples were taken out of the original packages filled with the physiological saline solution (0.9% NaCl), rinsed 3 times with deionized (DI) water, and DI water was removed with the filter paper. When preparing the samples, care was taken to prevent the analytical area of the contact lens from touching the filter paper. To measure the roughness and the chemical composition of the surface, each lens was cut into a noncolored part (central clear zone, 1) and two colored parts, all with a size of about 5 mm × 5 mm. The two colored parts were taken from the front surface (2) and the back surface (3), as shown in Figure 1(a).

Figure 1(b) shows the five-step procedure to prepare the cross-sectioned contact lens samples for analysis using SEM-EDX and ToF-SIMS. Step 1: rinse a new lens in DI water. Step 2: place the lens on the filter paper and allow the filter paper to absorb the water. Step 3: to prevent the lens from breaking, freeze the lens surface by placing it in close proximity to liquid nitrogen and then immerse the lens in liquid nitrogen for 30 seconds. Step 4: take out the frozen lens and quickly press it with a hard and flat object. Step 5: among the cut pieces (as described above), pick up a clean piece of the colored part and fix it to a metal block with the carbon tape. The liquid nitrogen method was used because cutting with a knife could have caused compression of surface components or breaking of the pigment layer [28, 35].

### 2.2. Analytical Methods

The surface roughness was measured on the central clear zone and on the front and back surfaces of the colored part by AFM (XE-100, Park Systems, Suwon, Korea). AFM images were acquired under ambient conditions in the tapping mode. Areas of 20 *μ*m × 20 *μ*m were analyzed at three points per sample. The values of the root mean square roughness (*R*_rms_) and average roughness (*R*_a_) were calculated with XEI software supplied by Park Systems. Roughness results presented as mean ± standard deviation were applicable, and all data were analyzed using Excel 2013 software (Microsoft, Redmond, WA). Surface roughness was normally distributed and was compared using the unpaired *t*-test. SEM-EDX (Teneo VS, FEI, Hillsboro, USA) was used to evaluate the cross-sectional images of the cosmetic contact lenses. The images were recorded at an acceleration voltage of 10 kV using backscattered electron detection. To measure the depth of the color layer, 4,000× magnification images of each cross-sectioned sample were obtained. We also obtained quantitative information on the pigment and nonpigment layers of the cross section. ToF-SIMS analysis was performed using a TOF SIMS5 (ION-TOF GmbH, Münster, Germany). The TOF SIMS5 system was equipped with a Bi^+^ and Cs^+^ primary ion beam source. The surface spectra and surface 2D image were obtained in the high-current bunched mode using a 25 keV Bi_3_^+^ ion analysis beam, and cross-sectional 2D image mapping was obtained in the burst alignment mode. The 3D chemical mapping was performed by sputtering with Cs^+^ at 3 keV under a current of 25 nA. The spectra and 2D and 3D images were summarized using SurfaceLab 6.8 and 7.0.

## 3. Results and Discussion

### 3.1. Surface Roughness

**Figure 2:**
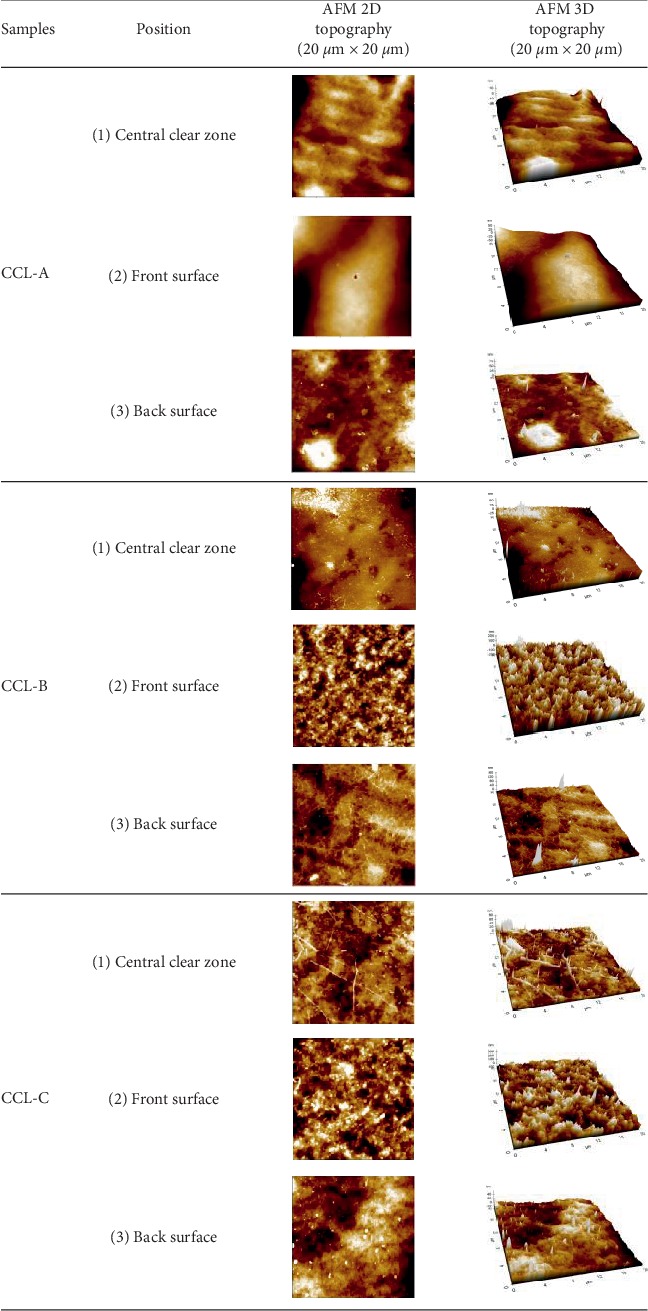
Comparison of surface topographies in images (20 *μ*m × 20 *μ*m) measured by AFM.

The surface roughness of lenses is known to affect the environmental conditions of the bacterial growth. The AFM observations of the lenses revealed different topographies depending on the manufacturer and the location of the analysis area, as shown in Figure 2.

Cosmetic contact lenses CCL-A, CCL-B, and CCL-C were imaged at the noncolored part (central clear zone (1)) and the front side (front surface (2)) and the eye-contact side (back surface, (3)) of the colored part, respectively. The topographic images revealed differences in the morphologies of lenses CCL-A, CCL-B, and CCL-C. Moreover, in lens CCL-A, the front surface of the colored part was highly curved. Lenses CCL-B and CCL-C had more roughly shaped colored parts overall.

Table 2 summarizes the roughness values and the statistical findings for each lens type in the different lens areas. Comparing the *R*_rms_ and *R*_a_ values showed that the roughness values were not significantly different between the central clear zone and the back surface of the colored part of the three products. All three products had the highest *R*_rms_ and *R*_a_ values on the front surface of the colored part. Lens CCL-A had relatively small values of *R*_rms_ and *R*_a_ roughness compared with lenses CCL-B and CCL-C. In lens CCL-A, the roughness values also showed a smaller difference between the colored (front surface) and noncolored (central clear zone) regions compared with the other lens types. The *R*_rms_ values for lens CCL-A ranged from 3 to 26 nm, while the *R*_rms_ values for lenses CCL-B and CCL-C ranged from 7 to 50 nm and from 5 to 43 nm, respectively. Lens surface roughness between different sides was compared using the unpaired *t*-test, as shown in Table 2. At the colored parts, all lenses with a surface pigment had significant difference of roughness between the front and back surfaces (*P* < 0.050 indicates statistical significance). The *R*_rms_ and *R*_a_ for the central clear zone and the back surface of lens CCL-B were not significantly different (for *R*_rms_, *P*=0.1860; for *R*_a_, *P*=0.1308). However, the *R*_rms_ and *R*_a_ for the front and back surfaces in the colored part were significantly different (for *R*_rms_, *P*=0.0004; for *R*_a_, *P*=0.0003). The relatively low roughness of lens CCL-A can be attributed to the fact that the pigment layer was located deeper beneath the surface while the pigment layers of lenses CCL-B and CCL-C were more exposed to the surface. These observed results are shown in Section 3.2. The above findings of the roughness of cosmetic contact lenses combining pigment layers are in agreement with the literature [21, 23]. The differences between manufacturers can be mainly attributed to the fabrication process of the pigment layer and the location of the pigment layer in the lenses.

### 3.2. Cross-Sectional Structures and Components

**Figure 3:**
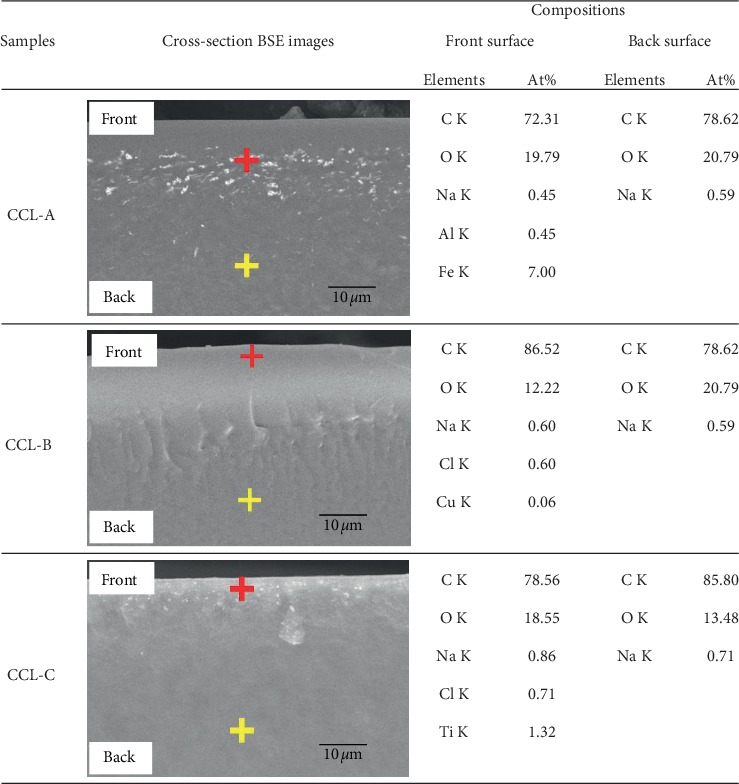
Cross-sectional BSE images and analyses of compositions by SEM-EDX. The crosses represent the EDX analysis points, with red crosses for colored layer points and yellow crosses for the noncolored layer.

The cross-sectional structure of the colored part of the lens, which had previously been fractured by freezing with liquid nitrogen, was observed in backscattered electron (BSE) images of SEM at a magnification of 4000× and subjected to elemental characterization by EDX. In the BSE images, the intensity contrast differed between samples as shown in Figure 3, indicating the presence of different types of materials.

The difference in contrast between the various areas in the SEM-EDX image of each contact lens was used to detect the chemical composition. The cross marks in Figure 3 represent the EDX analysis points, in which a red cross is a pigment layer and a yellow cross is a pigment-free layer. In the EDX analysis, we were careful to protect the lenses against high-voltage exposure and to reduce the damage to the samples as far as possible during the observations. In lens CCL-A, the metallic components Al and Fe were detected several microns below the surface. The SEM images showed no pigment particles on the top of lens CCL-A, while a particle layer was seen on the surface of both the other lenses. The pigment particles in lens CCL-A were buried below the front surface of the lens at an average depth of 4.4 *μ*m. In the lenses CCL-B and CCL-C, metallic components were observed near the surface, and in particular, Cu and Cl were detected in lens CCL-B, while Ti and Cl were detected in lens CCL-C.

**Figure 4:**
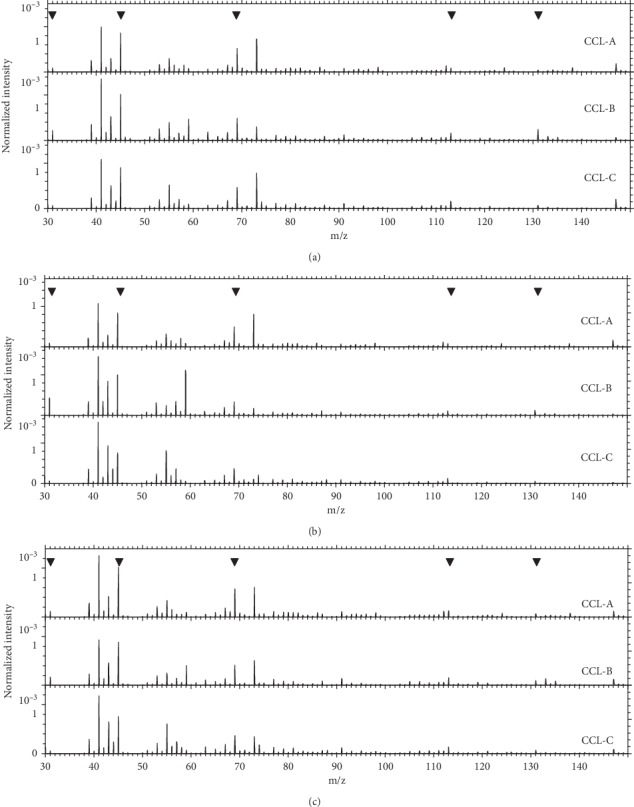
Positive-ion ToF-SIMS spectra of cosmetic contact lens revealed the presence of the bulk polymer PHEMA at all surfaces of three lenses. (a) Noncolored part: (1) central clear zone. (b) Colored part: (2) front surface. (c) Colored part: (3) back surface.

Figures 4(a)–4(c) show the positive-ion ToF-SIMS spectra and revealed the presence of the bulk polymer PHEMA on the central clear zone of the noncolored part and the front surface and the back surface of the colored part for the three different brown contact lens samples. A series of oxygen-containing hydrocarbons from HEMA was detected, such as CH_3_O^+^ (*m*/*z* 31.02), C_2_H_5_O^+^ (*m*/*z* 45.03), C_4_H_5_O^+^ (*m*/*z* 69.03), C_6_H_9_O_2_^+^ (*m*/*z* 113.06), and C_6_H_11_O_3_^+^ (*m*/*z* 131.07), along with pigments and contaminants, such as poly(dimethylsiloxane), dioctyl phthalate, sodium, aluminum, potassium, titanium, iron, and copper, which are primarily derived from the various processing steps in lens manufacture.

**Figure 5:**
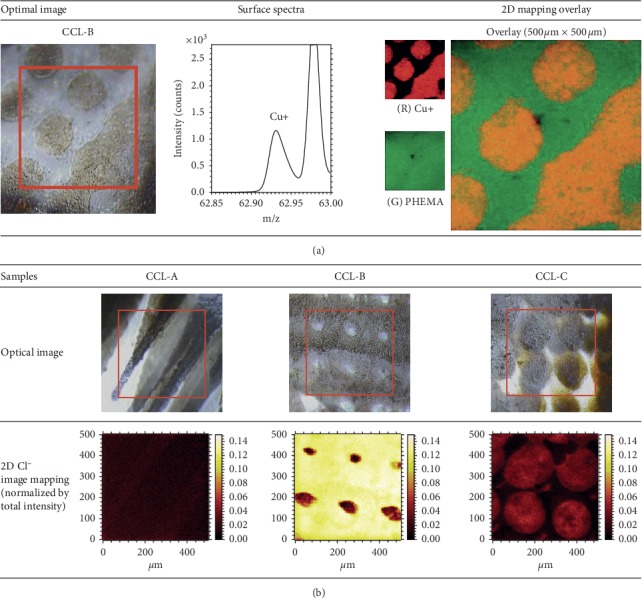
Optical images of the analysis area and ToF-SIMS 2D image mapping on the front surface of the colored part. (a) Overlay image of Cu^+^ and PHEMA for CCL-B. (b) Cl^−^ mapping images normalized by total intensity for lenses CCL-A, CCL-B, and CCL-C.

The positive ion spectra of lens CCL-A did not show any metal ions from the surface of the central clear zone and the front surface of the pigment layer, even though Fe was observed in the EDX analysis. This result supported that the metallic pigment layer of the lens CCL-A was located a few *μ*m deeper as detected by EDX. The front surface of lens CCL-B showed an intense copper peak in the EDX analysis, while the central clear zone did not. The ToF-SIMS spectra and the 2D mapping image for the front surface of lens CCL-B are shown in Figure 5(a). The Cu^+^ image mapping is displayed in red, and the combined image of PHEMA components is displayed in green. Thus, these results also clearly indicate that the metallic pigment layer of lens CCL-B is located on the surface. In the ToF-SIMS spectrum of lens CCL-C, the Ti peak was not observed in the colored part, despite the observation of Ti in EDX analysis. The pigment layer might have been located slightly beneath the lens surface, beyond the sampling depth of ToF-SIMS (>3 nm). In Figure 5(b), a Cl peak was observed only in the colored part of lens CCL-B and lens CCL-C, as shown in SEM-EDX results. The presence of a Cl peak suggested the presence of chloride compounds in the pigments containing chlorine.

**Figure 6:**
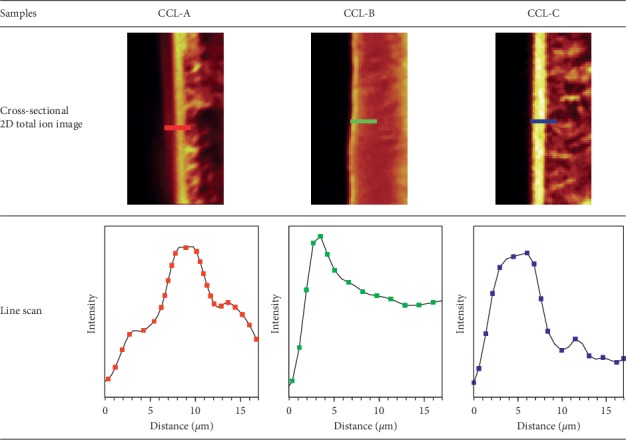
ToF-SIMS results of cross-sectional 2D image mapping and line scan of the surface layer for lenses CCL-A, CCL-B, and CCL-C.

Next, ToF-SIMS mapping was performed using the cross-sectioned samples. The fairly flat surface of the cross-sectioned sample obtained by a new method provides the clear ToF-SIMS image to confirm the position of the pigment layer. Strongly ionized materials typically appear bright in a total ion mapping image [32, 35].  Figure 6 shows the line profile of each bright layer for each cross-sectioned lens sample. For lens CCL-A, the bright layer appeared to be covered by another layer between it and the top surface, which indicated that the pigment layer was beneath the surface. For lens CCL-B, the bright layer appeared near the top surface and was much thinner than that of lens CCL-C. This observation confirms the previous SEM-EDX and ToF-SIMS image mapping results concerning the position of the pigment layer on the outer surface for lens CCL-B. The ToF-SIMS image mapping of the cross-sectioned lens was successfully achieved, which was not possible previously because of the tricky sample preparation. The different pigment locations result in each lens having its own characteristic properties, and these different locations presumably arise from variations in the manufacturing processes.

### 3.3. 3D Chemical Distributions

Further, the acquisition of ToF-SIMS images was challenging to obtain the 3D chemical distribution in specific regions of the cosmetic contact lenses. By sputtering with a Cs^+^ ion beam and analyzing the lens sample with a pulsed Bi_3_^+^ ion beam, component ions can be detected simultaneously and reconstructed in 3D distribution. However, in the case of polymer materials, the sputtering process generates various fragments and it can be challenging to determine where the fragments originated from for a particular substance. Nonetheless, even when the exact identity of a substance is not known, different substances can be distinguished by investigating the 3D component ion distribution. The chemical identities of the polymers constituting these contact lenses differ depending on the manufacturer, and the detailed composition of the pigment layers is not publicly known. The positions of the colored layers were determined by identifying the spatial distribution of the polymer and metal pigment fragments. The 3D chemical distribution of the colored part provides very valuable information on the spatial distribution of the pigment layer.

**Figure 7:**
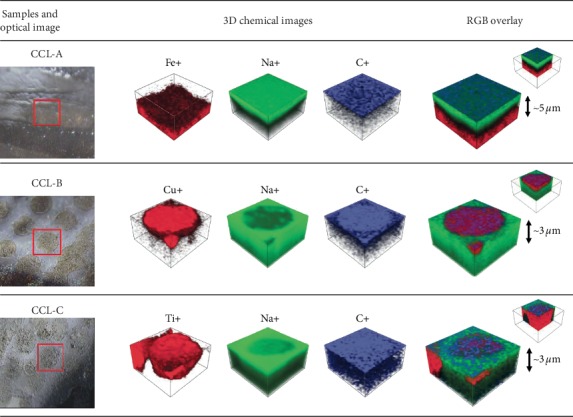
Optical images of the analysis area and 3D chemical images by ToF-SIMS.

Figure 7 shows optical images and 3D chemical images for the surface of each lens. In the optical images, the red square indicates the area analyzed by ToF-SIMS. Lens CCL-A was analyzed in the region where the pigment layer was uniform. Lens CCL-B and lens CCL-C were analyzed in a region where the pigment layer consisted of circular deposits of the pigment rather than a uniform layer. For lens CCL-A, as expected from the SEM images and EDX results, the Fe metal component from the pigment layer was positioned at around 3−4 *μ*m below the surface and is displayed in red. The green and blue parts are presumed to be polymers containing Na^+^ and C^+^, respectively, and these materials appear in all the samples although in different locations depending on the lens type. In lens CCL-B, the 3D chemical distribution of the pigment layer was represented by peaks of Cu at *m*/*z* 62.94 and 64.93, as can be seen directly at the front surface in the circular pattern region. Finally, in lens CCL-C, the pigment layer was represented by peaks of Ti at *m*/*z* 45.95, 46.95, and 47.95 and was visible immediately below the surface. Thus, the metallic color components of the pigment layers can be monitored spatially by a 3D ion image. Thanks to the ToF-SIMS analysis, it was possible to acquire the 3D chemical distribution of the cosmetic contact lenses including the pigment layer of interest.

## 4. Conclusions

AFM, SEM-EDX, and ToF-SIMS were used to investigate three kinds of brown cosmetic contact lenses. AFM measurements of the surface topographies and the *R*_rms_ and *R*_a_ roughnesses showed that the colored parts were rougher on the front surface than on the back surface in all cases. The position and distribution of the pigment layer were measured by SEM and were different for each product. The pigment layer of lens CCL-A was located a few microns below the front surface, while the colored layers of lenses CCL-B and CCL-C were exposed to the front surface. EDX analysis of the composition showed different metals in each pigment layer. In the ToF-SIMS results, the surface spectra of lens CCL-A were similar in all analyzed regions, but lenses CCL-B and CCL-C revealed distinct peaks in the colored part. In the 2D ToF-SIMS analysis of the colored part of the lens samples prepared by a new method, a group of fragment ions with characteristic distributions was successfully found, but it was difficult to accurately identify the fragments. Although further research is needed on the materials that make up cosmetic contact lenses, the ToF-SIMS technique in combination with other analytical methods, such as AFM and SEM-EDX, is a promising approach to explain the properties of color contact lenses in terms of their elemental distribution and surface structure. The 3D ToF-SIMS chemical imaging of cosmetic contact lenses is of particular importance as it provides an effective, full through-thickness chemical characterization of pigment layers. The 3D ToF-SIMS ion images of the colored part show the spatial chemical distribution of pigment layers and can be used to determine the pigment elution. Therefore, the methodological development and application of the ToF-SIMS technique to lens relevant industry will be useful for making a new way to evaluate the safety of cosmetic contact lenses.

## Figures and Tables

**Table 1:** Properties of the cosmetic contact lenses.

Sample name	Color	Lens image	Materials	Manufacturer	BC (mm)/dia (mm)	FDA group	Water content (wt.%)
CCL-A Acuvue	Radiant bright	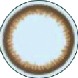	Etafilcon A	Johnson & Johnson Vision care	8.5/14.2	IV	58
CCL-B Bausch + Lomb	Crystal brown	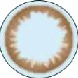	HEMA + NVP	Bausch + Lomb	8.6/14.2	I	55
CCL-C Clalen	Latin	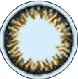	Metafilcon A	INTEROJO	8.6/14.2	IV	55

**Table 2:** Atomic force microscopic evaluation for root mean square roughness (*R*_rms_) and average roughness (*R*_a_) of the brown cosmetic contact lens at each area.

Samples	Position	*R*_rms_ (nm) (mean ± SD)	*P* value^a^ for *R*_rms_	*R*_a_ (nm) (mean ± SD)	*P* value^a^ for *R*_a_

CCL-A	(1) Central clear zone	3.57 ± 1.15	Clear: front	2.72 ± 0.82	Clear: front
			0.0015		0.0014
	(2) Front surface	26.19 ± 4.97	Front: back	21.03 ± 3.90	Front: back
			0.0017		0.0014
	(3) Back surface	3.75 ± 1.42	Back: clear	2.67 ± 1.02	Back: clear
			0.8702		0.9564

CCL-B	(1) Central clear zone	7.40 ± 0.22	Clear: front	5.40 ± 0.41	Clear: front
			0.0002		0.0002
	(2) Front surface	49.98 ± 5.64	Front: back	39.35 ± 4.52	Front: back
			0.0004		0.0003
	(3) Back surface	9.86 ± 2.66	Back: clear	7.05 ± 1.45	Back: clear
			0.1860		0.1308

CCL-C	(1) Central clear zone	5.12 ± 0.42	Clear: front	3.72 ± 0.54	Clear: front
			0.0001		0.0001
	(2) Front surface	43.17 ± 4.64	Front: back	33.44 ± 3.31	Front: back
			0.0002		0.0001
	(3) Back surface	8.34 ± 0.23	Back: clear	6.46 ± 0.19	Back: clear
			0.0003		0.0012

*R*_rms_: root mean square roughness; *R*_a_: average roughness; SD: standard deviation. ^a^Comparison of lens surface roughness between different sides using the unpaired *t*-test. At the colored parts, all lenses with the surface pigment had significant difference of roughness between the front and back surfaces (*P* < 0.050 indicates statistical significance).

## Data Availability

The data used to support the findings of this study are included within the article and also available from the corresponding author upon request.
